# An observational multi‐clinic implementation science study to evaluate barriers and enablers of real‐world use of confirmatory blood‐based biomarkers for the diagnosis of early Alzheimer’s disease

**DOI:** 10.1002/alz.088784

**Published:** 2025-01-09

**Authors:** Leah Zullig, Soeren Mattke, Jo Vandercappellen, Daryl Jones, Harald Hampel, Richard Batrla

**Affiliations:** ^1^ Duke University School of Medicine, Durham, NC USA; ^2^ University of Southern California, Los Angeles, CA USA; ^3^ Eisai Inc., Nutley, NJ USA; ^4^ Eisai Inc, Nutley, NJ USA

## Abstract

**Background:**

New innovations take a long time to be utilized into routine healthcare due to intrinsic and practical barriers. Implementation science can identify such barriers and offer potential solutions to speed up this process. Blood‐based biomarkers (BBBMs) may enable scalable confirmation of amyloid pathology in the Alzheimer’s disease (AD) care pathway with test performance similar to cerebrospinal fluid (CSF) testing and positron emission tomography (PET), so‐called confirmatory biomarkers as they can corroborate amyloid pathology with high certainty. However, establishing effectiveness does not guarantee translation into the care pathway. The objective of this study is to evaluate the feasibility of implementing BBBMs as confirmatory diagnostic tools for early AD in real‐world clinical practice.

**Method:**

This prospective, multi‐clinic study will use the updated Consolidated Framework for Implementation Research to guide the implementation strategy. Consenting participants with first referral for evaluation of progressive cognitive decline will be assigned to: Arm 1 (standard‐of‐care [SOC]) or Arm 2 (confirmatory BBBM). Laboratory, cognitive and magnetic resonance imaging assessments will be performed in both arms as per the SOC in a memory clinic where BBBMs are accessible. Amyloid biomarkers will be confirmed by PET or CSF assessment in Arm 1 and confirmatory BBBMs in Arm 2. Implementation science questionnaires (clinicians) and qualitative interviews (clinicians and patients) will be conducted at baseline and at end of study in both arms.

The following implementation science outcomes will be evaluated: feasibility, acceptability and appropriateness of BBBMs using validated questionnaires; proportion of patients and reach (diversity representativeness [e.g., sex/race/age]) with a biomarker‐confirmed diagnosis; barriers and facilitators of BBBM implementation; time, number of visits and costs for a biomarker‐confirmed diagnosis. An estimated 60 subjects will participate in qualitative interviews based on information saturation guiding principle.

**Result:**

The study will generate real‐world data on implementation of confirmatory BBBMs into clinical practice, specifically into a memory clinic set‐up. Interim results will be presented as available.

**Conclusion:**

This study will provide a rationally and systematically tested and studied blueprint and recommendations for implementing confirmatory BBBMs into healthcare systems by identifying facilitators and barriers. The outcomes are intended to be used in educational programs for accelerated uptake of BBBMs.

